# Non-Destructive Optical Monitoring of Grape Maturation by Proximal Sensing

**DOI:** 10.3390/s101110040

**Published:** 2010-11-09

**Authors:** Naïma Ben Ghozlen, Zoran G. Cerovic, Claire Germain, Sandrine Toutain, Gwendal Latouche

**Affiliations:** 1 CNRS, Laboratoire Écologie, Systématique et Évolution, UMR 8079, Bât. 362, Orsay, Université Paris-Sud, Orsay, AgroParisTech, Paris, F-75231, France; E-Mails: naima.ghozlen@u-psud.fr (N.B.G.); gwendal.latouche@u-psud.fr (G.L.); 2 CIVC, 5 rue Henri-Martin, boîte postale 135, F-51200, Epernay, France; E-Mail: claire.germain@civc.fr (C.G.); 3 Champagne Moët & Chandon, 20, Avenue de Champagne, F-51200, Epernay, France; E-Mail: stoutain@mhws.fr (S.T.)

**Keywords:** Pinot Noir, Pinot Meunier, Chardonnay, phenolic maturity, anthocyanins, chlorophyll fluorescence, fruits, vegetables, ripening

## Abstract

A new, commercial, fluorescence-based optical sensor for plant constituent assessment was recently introduced. This sensor, called the Multiplex^®^ (FORCE-A, Orsay, France), was used to monitor grape maturation by specifically monitoring anthocyanin accumulation. We derived the empirical anthocyanin content calibration curves for Champagne red grape cultivars, and we also propose a general model for the influence of the proportion of red berries, skin anthocyanin content and berry size on Multiplex^®^ indices. The Multiplex^®^ was used on both berry samples in the laboratory and on intact clusters in the vineyard. We found that the inverted and log-transformed far-red fluorescence signal called the FERARI index, although sensitive to sample size and distance, is potentially the most widely applicable. The more robust indices, based on chlorophyll fluorescence excitation ratios, showed three ranges of dependence on anthocyanin content. We found that up to 0.16 mg cm^−2^, equivalent to approximately 0.6 mg g^−1^, all indices increase with accumulation of skin anthocyanin content. Excitation ratio-based indices decrease with anthocyanin accumulation beyond 0.27 mg cm^−2^. We showed that the Multiplex^®^ can be advantageously used in vineyards on intact clusters for the non-destructive assessment of anthocyanin content of vine blocks and can now be tested on other fruits and vegetables based on the same model.

## 1. Introduction

It is now well accepted that premium wine quality depends on the quality of the grapes used to produce it. Winemakers commonly have a target ripeness for the fruit according to the wine they want to produce. Pinot Noir intended for champagne sparkling wine production, will have a very different ripeness target compared to that for Pinot Noir still wine. Lower sugar, higher acidity and more neutral flavours are desired for sparkling wine compared to still wine [1], so “ripeness” and harvest occur earlier for sparkling wine. There are other non-compositional factors that influence the decision to harvest, including labour availability, tank space limitations, seasonal changes such as rainfall and heat waves and other factors beyond the winemaker’s control. Because the climate during the growth season is one factor beyond the winemaker's control, very different outcomes occur from year to year that influence the decision to harvest [2–4]. Therefore, increased efforts are invested to accurately estimate grape maturation kinetics and the half-véraison stage in order to predict the best harvest date [5], to define homogenous maturation (quality) zones [6] and to select grapes at the weighing bridge [7]. Even in Champagne, where white wine is primarily produced, there is an increasing trend towards rosé champagne and, therefore, an increasing need for quality red wine. Champagne producers have the advantage of being able to mix red and white wines to produce rosé.

Among the various grape constituents, sugar content, pH and acidity levels are the most frequent cluster characteristics used to assess ripeness (technical maturity) [5]. Sugar levels appear to be fairly uniform across the population of berries, displaying a coefficient of variation (CV) around 3% [8]. For red grape varieties, both technical maturity and phenolic maturity were found to be of paramount importance, but phenolic maturity was much more variable across the vineyard (CV = 14%) [8]. Phenolic maturity can be assessed by measuring either total phenolics or skin anthocyanin content, which is well correlated with total phenolics [9–11] but, more importantly, which reflects “smoother” skin phenolics that are preferred to seed proanthocyanidins.

The assessment of grape maturity in a vineyard block is performed by analysing representative samples of berries or grapes in the laboratory by standard wet chemistry analytical methods: hydrometry, refractometry, titration and spectrophotometry on extracts obtained at regular time intervals [5,7,12]. Although new analytical techniques, such as HPLC, have been introduced for a more precise estimation of the phenolics [13] or the NIRS of less-refined samples, to decrease the time needed for analysis [14], laboratory analysis is still the bottleneck for the proper estimation of the grape status of the vineyard. The representativeness of the berry samples is the second major concern.

Techniques based on intrinsic fruit fluorescence (autofluorescence) have been successfully applied to grapes [15–19] and apples [20,21]. Fluorescence indices, based on the comparison of chlorophyll fluorescence excited at two wavelengths, were shown to reflect not only the content of epidermal phenolics in leaves [22,23], but also olive [24] and grape berry [16] skin anthocyanin content. The method is often called the chlorophyll fluorescence screening method (cf. [16]) to distinguish it from the use of variable chlorophyll fluorescence linked to photosynthesis in leaves but also used on fruits [25,26]. Because of the use of a logarithm according to the Beer-Lambert law, the method is also called logFER (for logarithm of the fluorescence excitation ratio) [27,28]. Although the method provides satisfactory results, the different fluorescence-based indices for anthocyanins assessment have to be compared because they are based either on signal ratios [16,29] or on transformed single signals [18,19] that each have different advantages and drawbacks. In our previous works, we used a prototype version of a portable optical sensor with a different optical head geometry [18,19] than the one used in the present study. An industrial version is now commercially available under the same name Multiplex^®^ that includes both options of using the chlorophyll fluorescence screening method and the fluorescence emission ratios. There is thus a need to test its potential and limits for assessing grape phenolic maturity.

The objectives of this work were to validate the use of Multiplex^®^ indices based on the chlorophyll fluorescence screening method by: (1) calibrating the sensor’s different indices for the estimation of grape anthocyanin content, (2) producing a model to separate the decrease of green berries number from anthocyanin accumulation during maturation and (3) proposing and testing a protocol for the implementation of the sensor to Champagne conditions and grape varieties.

## 2. Experimental Section

### 2.1. The Multiplex Sensor

**Figure 1.**
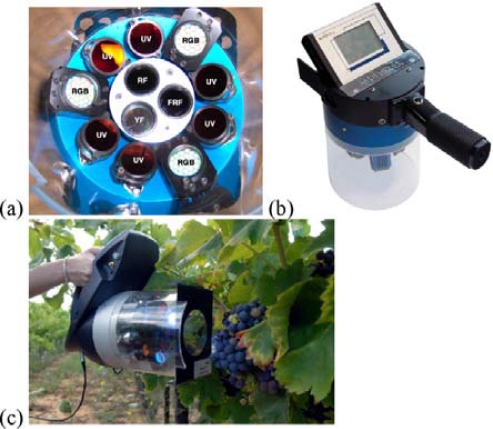
The Multiplex^®^ sensor. **(a)** Front view of the optical head with LED sources (6 UV & 3 RGB) and three detectors in the middle (YF, FRF, RF) identical for Multiplex^®^ 2 and 3. **(b)** Top view of the Multiplex^®^ 2 sensor showing the touch-screen interface and triggering button. **(c)** Measurement in the field with the Multiplex^®^ 3. For nomenclature, see Table 1.

Multiplex^®^ (FORCE-A, Orsay, France, patent pending) is a hand-held, multi-parametric fluorescence sensor based on light-emitting-diode (LED) excitation and filtered-photodiode detection that is designed to work in the field under daylight on leaves, fruits and vegetables (Figure 1).

**Figure 2.**
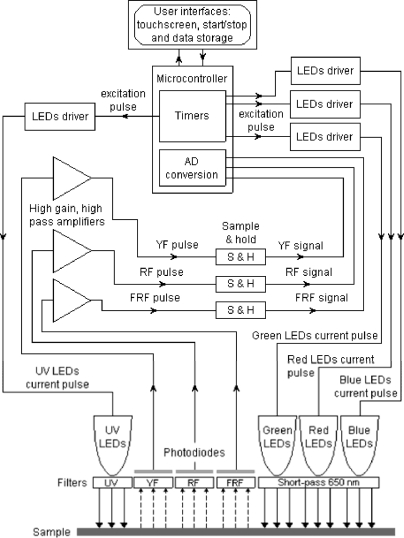
Block diagram of the Multiplex^®^ sensor.

A block diagram of Multiplex^®^ functions is shown in Figure 2. The present version of the sensor has a yellow (Y) filter at the third emission channel (it may also have a blue filter for blue-green fluorescence, BGF, according to FORCE-A). The sensor has six UV-light sources (LED-matrices) at 375 nm protected by DUG11 filters (Schott, Mainz, Germany) and it has three, Red-Blue-Green (RGB) LED-matrices emitting lights at 470 nm (blue, B), 516 nm (green, G) and 635 nm (red-orange, R) protected by a 650-nm short-pass filter (Edmund Scientific, United Kingdom) (Figure 1). LEDs are pulsed sequentially at 476 Hz with 20 μs per flash.

There are three, synchronised, photodiode detectors for fluorescence recording: yellow (YF), red (RF) and far-red (FRF), which are defined by the 590NB10, 678WB22 and 750WB65 interference filters (Intor, Socorro, NM USA), respectively. In addition, the RF channel has a 3-mm RG665 red glass filter (Schott, Mainz, Germany) and the FRF channel has a 3-mm RG9 far-red glass filter (Schott, Mainz, Germany). The sensor illuminates an 8-cm-diameter surface (50 cm^2^) at a 10-cm distance from the source and detector, where the cluster or berry plates were positioned for measurements, which lasted less than a second per cluster. Each measurement consisted of a train of 500 flashes of four colours (UV, B, G and R). The sensor calculates a set of chosen ratios after each series of four-color flashes. The mean and standard deviation of the 500 measurements for the 12 signals (Table 1) and 10 ratios are recoded on a SD card and displayed on the sensor’s screen [Figure 1(b)]. The Multiplex^®^ was used in the field under daylight and was also used indoors on berries. Two versions of the Multiplex were used that differed only in their design and ergonomics. Multiplex^®^ 2 [Figure 1(b)] was used for calibration, laboratory measurements and in the Fort Chabrol vineyard, and Multiplex^®^ 3 [Figure 1(c)] was used in commercial vineyard blocks.

Because the fluorescence screening method used in the Multiplex^®^ was described in detail in [16], here we will only identify and describe the nomenclature of the fluorescence indices provided in the commercial Multiplex^®^ sensors used in the present study. The decadic logarithm of the ratio of far-red fluorescence (FRF) excited at two different wavelengths [red FRF_R and green FRF_G (Table 1)] is called ANTH_RG because it is proportional to skin anthocyanin content:

(1) ANTH_RG=log(FRF_R/FRF_G)

We also calculated two equivalent indices based on the combination of blue and red excitation and blue and green excitation (not recorded on the sensor) that were tested in this study:

(2) ANTH_RB=log(FRF_R/FRF_B)

(3) ANTH_BG=log(FRF_B/FRF_G)

Ben Ghozlen *et al.* [19] recently proposed the use of a log-transformed version of an inverted, single-signal FRF_R:

(4) FERARI=log(1/FRF_R)=−log(FRF_R)

They found that the log-transformed version had a good positive correlation with skin anthocyanin content. This index is provided in the commercial Multiplex^®^ under the abbreviation FERARI (Fluorescence Excitation Ratio Anthocyanin Relative Index, used hereafter).

The emission ratio (SFR_R) is linked to the chlorophyll content of leaves [30,31] and grape berries [19]. It is a simple chlorophyll fluorescence ratio (SFR) of far-red emission (FRF, 735 nm) divided by red emission (FR, 685 nm) under red excitation:

(5) SFR_R=FRF_R/RF_R

Due to the overlap of the chlorophyll absorption and emission spectrum, re-absorption occurs at shorter wavelengths (RF) but not at longer wavelengths (FRF) [30,31]. Therefore, SFR increases with increasing sample chlorophyll content. It should be noted that the inverse ratio, RF/FRF, is also often used in the literature (cf. [32]). The latter will decrease with increases in chlorophyll content.

### 2.2. Multiplex Measurement Protocols

#### 2.2.1. Calibration with Standards

In order to compare the data obtained with other sensors and data collected under other measuring conditions, all Multiplex^®^ signals were standardised by dividing them by the values obtained on a blue plastic-foil standard (Force-A, Orsay, France) under exactly the same measurement conditions. The result was also corrected for temperature variations using a temperature response curve for each signal (calibrated in a temperature-controlled chamber from 10 to 45 °C). The blue standard has fluorescent properties similar to that of a leaf without flavonols or anthocyanins present.

**Figure 3.**
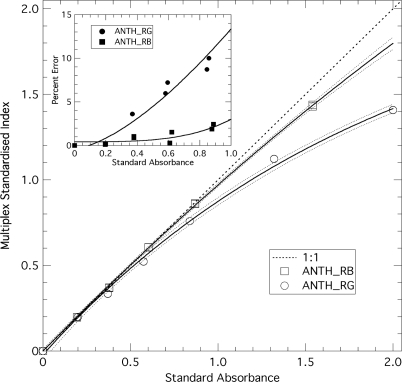
Calibration of the anthocyanins-related Multiplex^®^ indices using standards of known absorbance. Multiplex^®^ indices ANTH_RB and ANTH_RG were calculated from their respective signals [Equations (1) and (2)] and plotted on the y-axis. Best exponential fits (full lines) and 95%-confidence intervals of the fit (broken lines) are shown. Insert: percent error (difference) between standard absorbance and Multiplex^®^ derived absorbance.

To check the linearity of the ANTH Multiplex^®^ index, seven sheets of plastic, coloured filters of known transmittance (Force-A, Orsay, France) were layered in sequence above the blue standard. Before each new sheet was added, the stack was measured with the Multiplex^®^. The sequence was repeated by withdrawing the sheets (absorbance standards) one by one until only the blue fluorescence standard remained. The standard absorbances used for calibration (x-axis on Figure 3) were the differences between absorbances at 635 and 516 nm, and at 635 and 470 nm, for ANTH_RG and ANTH_RB, respectively.

For the test of sensitivity and detection limit, isolated chlorophyll-protein complexes of 0.3 mg mL^−1^ were added in 10-μL aliquots to 1 L of deionised water (5-cm water column). The bottom of the 1-L recipient was sitting on the opening of the Multiplex and was thus at the standard measuring distance (the sensor had its side facing up).

#### 2.2.2. Experimental Site and Sampling for Calibration

The study was conducted from mid-July to October 2008 in the Fort Chabrol vineyard in Epernay, France (Log. 03°57′ E, Lat. 49°02′ N) (cf. supplementary Figure S1). During this period, clusters of the red cultivars Pinot Noir (PN) and Pinot Meunier (PM), as well as the white cultivar Chardonnay (CH), were marked and measured on the sun-exposed face once or twice per week (15 dates) with the Multiplex^®^. Forty-two clusters were selected per cultivar, with 18 of them located at the first position on the mid cane. The other 24 clusters were chosen on four vines, with six clusters per vine and two clusters per each proximal, middle and distal cane. At each date, each cluster was measured only once to avoid the accumulation of variable chlorophyll fluorescence effects. The clusters were measured with the Multiplex front-piece pressed against the cluster. In parallel, 2 kg of clusters were sampled twice per week from the same block to perform technological analysis of the juice: pH, total acidity and sugar content.

#### 2.2.3. Vineyard Block Measurements and Sampling

Maturation of 40 commercial vineyard blocks from the Champagne region was followed twice per week by sampling 200 berries that were measured with the Multiplex^®^ 2 immediately before standard wet chemistry analyses: pH, total acidity, sugar content and anthocyanin content. In parallel, an additional measurement on 100 clusters was performed in the field with Multiplex^®^ 3 on a selection of 14 blocks, using seven blocks during the whole season (six to eight dates) and using seven blocks only twice before harvest (n = 53).

#### 2.2.4. Measurements on Berries in the Laboratory

For the calibration of the *P*-model (the contribution of red and green berries to Multiplex^®^ indices, see Section 3.3. hereafter), a non-fluorescent, black tray was completely filled with green berries and measured with the Multiplex^®^. Four measurements were taken along the tray of green berries, then red berries were progressively introduced in steps of 10% and the Multiplex^®^ measurements were repeated (Figure S2). For simplicity, we will call all berries having anthocyanins (whether red, purple or blue) “red berries” regardless of their state of maturity. For the validation of the combined *P*-model and *A*-model (the increase in skin anthocyanin content, see Section 3.4. hereafter), a visual estimation of the proportion of red berries (*p*) on 200-berry samples was performed from the photographs (Figure S2).

To test measuring distances, the tray was filled with three groups of berries (green, red and purple) (Figure S2). Each area was measured by the Multiplex^®^ at four different distances from the sample (11, 12, 13 and 14 cm).

#### 2.2.5. Preparation of Berry Skins

Clusters of PM and PN were sampled at four dates in August and September (day of year—DOY 226, 233, 240 and 261) for the calibration of the *A*-model. For each cultivar, berries were first measured individually and then grouped (19 berries) based on similar values of Multiplex^®^ indices for anthocyanins. Multiplex^®^ measurements were again performed on the 19-berry samples with berries grouped and oriented alike in a cluster on a special, perforated black plate (Figure S2). Berries were then frozen and kept at −80 °C. The upper half (flower scar side) of each of the 19 frozen berries was peeled off after partial defrosting. Refrozen skins were ground in liquid nitrogen 3 × 20 s (ball mill MM301, Retsch, Haan, Germany) and the obtained powder was stored again at −80 °C until anthocyanins extraction was performed.

### 2.3. Wet Chemistry

#### 2.3.1. Extraction and Estimation of Anthocyanins from Berry Skins

**Figure 4.**
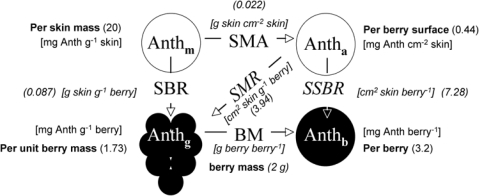
Four ways to express anthocyanin (Anth) content. Typical values for a mature 2-g Pinot Noir berry are indicated between round brackets and dimensions between square brackets. The conversion factors are SBR, skin-mass to berry-mass ratio, SMA, skin mass per area (fresh weight), BM, berry mass, SSBR, skin-surface to berry ratio (4.836 [BM/1.0817]^0.6667^), SMR, berry surface-to-mass ratio (4.836 [BM/1.0817]^−0.3333^). The value “1.0817” is the density of a berry having 20 °Brix. For an average molar mass of 500 gmol^−1^, 1 mg anthocyanins = 2 μmol, so all units can be transformed into μmols by multiplying by 2. Note that SBR = SMA × SMR and SSBR = SMR × BM.

Anthocyanins were extracted according to the method of Pirie and Mullins [33] with modifications. Skin powder, 10 to 50 mg, was transferred to 9 mL of acidified extraction solvent (MeOH/H_2_O/HCl 12N, 50:49:1, v/v/v) and stirred in glass tubes (stirring module Reacti-Therm III, Pierce, Paris, France) for 45 min in the dark at room temperature. Samples were then centrifuged for 10 min at 4,100 g. The absorbance spectra of supernatants were measured immediately upon extraction on a spectrophotometer (HP 8453; Agilent, Les Ulis, France) from 190 to 1,100 nm. Anthocyanins content was expressed in equivalents of malvidin-3-*O*-glucoside (oenin, Extrasynthèse, Lyon, France) using a molar absorptivity of 28,500 M^−1^ cm^−1^ at 530 nm after subtraction of a residual absorbance at 780 nm [16]. A molar mass of 500 g mol^−1^ was used for conversions between molar and mass units. The average berry mass (BM) and fresh skin mass per area (SMA) was measured for each 19-berries sample. The SMA was obtained by weighing 12 skin discs of 5 mm diameter for each sample. The average volume and surface area were calculated by assimilating the berry to a sphere with a density of 1.0817 kg dm^−3^. The SMA of dry skins was also measured because it was much less variable than its fresh counterpart. We calculated fresh SMA from dry SMA using the average water content obtained per cultivar (CH, PM, PN) (around 72%). In Figure 4, we summarise the relationship among the four ways to express anthocyanin content in order to facilitate comparisons among the results obtained using different methods [optical, HPLC (literature data) and standard wet chemistry] and used in different contexts (physiology, ecology, oenology).

#### 2.3.2. Standard Wet Chemistry Analysis of Sampled Grapevine Blocks

For the estimation of sugar (glucose + fructose) content, two methods were used: hydrometry for the Fort Chabrol samples and refractometry for the commercial block samples. The results of both methods were converted into gL^−1^. Total acidity was measured by titration with bromothymol blue (Fort Chabrol) or with an automatic pH-meter (commercial blocks) and expressed in gL^−1^ of equivalent H_2_SO_4_ (1 gL^−1^ H_2_SO_4_ = 1.53 gL^−1^ tartaric acid).

For estimation of anthocyanins, each sample of 200 berries from the commercial blocks was ground in a kitchen blender (high speed, 1 min). Fifty grams of the slurry were heated for 30 min at 70 °C in a water-bath and then cooled at ambient temperature for 30 min (modified ITV method, [34]). After centrifugation at 4,000 rpm for 10 min, 0.4 mL of supernatant was added to 4 mL of an acidified, aqueous solution (H_2_O/HCl, 98:2, w/w). After a second centrifugation at 6,000 rpm for 10 min, the absorbance of the last supernatant was measured at 520 nm and the anthocyanin content per berry mass (mg g^−1^) was calculated according to [34].

### 2.4. Data Elaboration

Data were treated, transformed, statistically analysed, fitted and plotted using a combination of software: Excel 2003 (Microsoft, USA), Statistica 6 (StatSoft, Tulsa, OK USA) and Igor Pro 6.02 (WaveMetrics, USA). Model solving and computations were performed with Mathematica 4 (Wolfram Research, Champaign, IL, USA).

## 3. Results and Discussion

### 3.1. Standardisation and Sensitivity of Multiplex Sensor Indices

In addition to the intrinsic linearity of the response of Multiplex^®^ detectors guaranteed by the producer, we have tested the linearity of the ANTH indices themselves using standards of known absorbance. Figure 3 shows that ANTH_RG deviates substantially from the y = x line above the absorbance of 0.9 (more than 10%). ANTH_RB, corresponding to the ratio of red to blue excitation, is more linear and has less than 10% deviation up to the absorbance of 2. However, none of the ANTH values acquired in this study were larger than 0.9; we can thus consider them all having a linear response. The FERARI index, which is just a log transformation of FRF_R, was linearly related to the absorbance of the standards over two orders of magnitude (therefore, not presented in Figure 3). The addition of aqueous solution of chlorophyll-protein complexes revealed the detection limit of the sensor’s FRF_R signal to be 0.7 μg chlorophyll L^−1^ (3.5 ng chlorophyll cm^−2^) and the sensitivity to be 1.86 mV μg^−1^ chlorophyll (1 mV per 2.7 ng chlorophyll cm^−2^).

Table 2 shows the various sources of variability and the precision of the Multiplex^®^ measurements. Repeatability was calculated from 30 consecutive measurements on the blue standard at 25 °C. For repeatability, we present the signal both in millivolts and as the standardised signal because the latter was obtained using the same blue standard. In addition, presenting percentage standard deviation (%SD) for the standardised ANTH_RG index is mathematically meaningless because it is a logarithm of a signal ratio; therefore, its mean is equal to zero in the absence of anthocyanins. The precision of the Multiplex^®^ was good (better than 1%) and the reproducibility, assessed using the same type of measurement but during the entire season (n = 11) at a temperature range from 27 to 28 °C, was satisfactory (better than 2%). Temperature affected the signals (1% per °C) more than the ratios (0.25% per °C, Table 2).

In the most recent version of the Multiplex^®^ (the Multiplex^®^ 3 that we used in the second part of this study in the field), all of the signals were corrected for temperature in the sensor itself. The major source of variability was the distance of measurement. A 30 to 40% variation may be induced depending on the berry type for a 30% deviation from the standard distance of measurement (Table 2). As expected, a single signal (FRF_R) and its transformation as a FERARI index (cf. below) were influenced much more by the distance of measurement than an index based on either fluorescence emission (SFR_R) or excitation (ANTH_RG) ratio (Table 2), decreasing to only 2 to 4% depending on the berry type. Two conclusions can be drawn from the data in Table 2. First, a single measurement per cluster is sufficient. Repeated successive measurements would only increase the variability due to the induction of the variable chlorophyll fluorescence [26] because the latter depends on environmental conditions. Second, the FRF_R value for green berries was 40% larger than for the blue standard (1.373 *vs*. 1) and ANTH_RG was larger than zero (0.072). These results indicate that green berries are more fluorescent than the blue standard and they are less excited in the green than the standard (compared to red light excitation). Because this absorption might vary with chlorophyll content and berry structure, we did not correct for it (cf. negative value in Figures below).

### 3.2. Changes of Multiplex^®^ Indices during Grape Maturation

**Figure 5.**
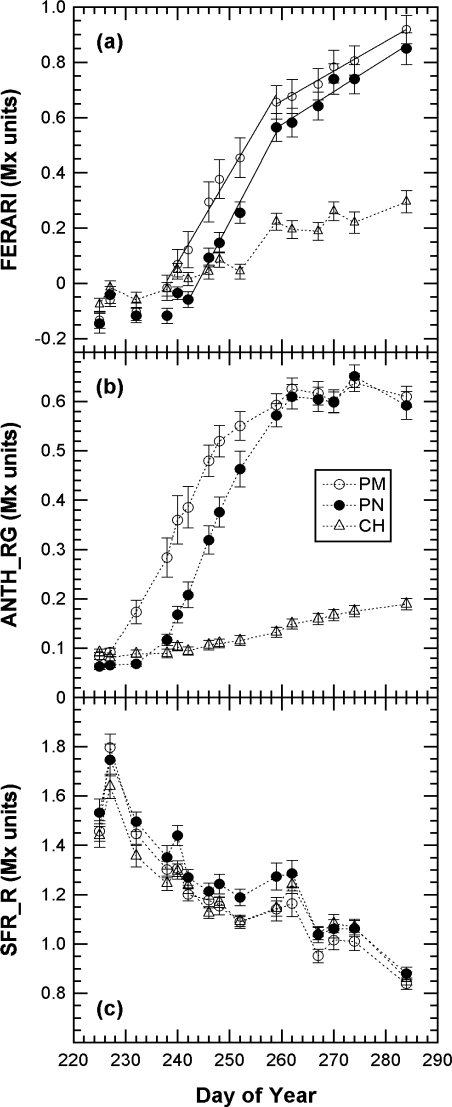
Time course of changed to the Multiplex^®^ indices during the 2008 season. Mean Multiplex^®^ index values for 40 marked clusters per cultivar for anthocyanins **(a,b)** and chlorophyll **(c)**. CH = Chardonnay, PM = Pinot Meunier, PN = Pinot Noir. Error bars are 95% confidence intervals. In (a) linear fits for FERARI for DOY 238–260 and for DOY 260–284.

We first present the kinetics of Multiplex indices produced by the sensor recorded on 40 marked clusters during the whole 2008 maturation season (Figure 5).

The kinetics were similar to the one obtained in 2007 with the Multiplex^®^ prototype [19], but here the sampling was more frequent (twice a week) and lasted 45 days longer, until mid October (DOY 284), when the harvest had been finished in all of Champagne (CIVC, personal communication). This timeframe allowed us to see that the FERARI index continued to increase until the last day. However, there was a change in slope from DOY 260 [Figure 5(a)] corresponding to the date from which ANTH_RG remained constant [Figure 5(b)]. DOY 260 corresponded to the date when all berries of all clusters were coloured red in Pinot Noir and Pinot Meunier (end of véraison, stage BBCH 85 [35]). Therefore, there were two types of potential influences on anthocyanin-related optical signals: the influence of the proportion of red to green berries and the effect of anthocyanin screening in red berries. We will analyse these two influences independently in the next sections.

**Figure 6.**
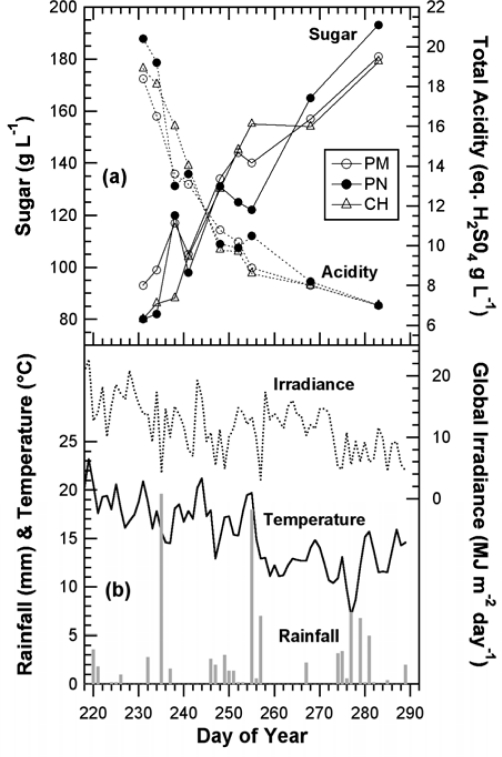
Characteristics of the Fort Chabrol vineyard block in 2008. **(a)** Changes in grape sugar and acidity during the season (technical maturation). **(b)** Weather data (Epernay weather station, France): daily rainfall (mm) and daily temperature (°C), left scale; global daily irradiance (MJ cm^−2^ day^−1^), right scale.

**Figure 7.**
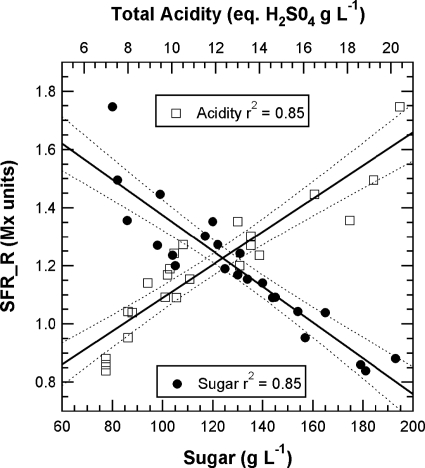
Correlation between the SFR chlorophyll index and technical maturity for all three cultivars. SFR was obtained by non-destructively following 40 marked clusters during the season. Sugar and acidity were measured (destructively) in the must of 2-kg samples of grapes from the same block.

The chlorophyll-related SFR index, whose calibration is beyond the scope of this paper, decreased steadily in all cultivars during grape maturation. Although technical maturity, estimated on 2-kg samples of grape from the same block, showed large fluctuations (Figure 6) probably caused by a sampling problem, a good correlation can be seen between SFR and both sugar and total acidity (r^2^ = 0.85 and r^2^ = 0.85, respectively) (Figure 7).

This characteristic could be especially useful for the non-destructive, optical monitoring of white grape cultivars devoid of anthocyanins. In contrast, ANTH_RG increased steadily during the whole season [Figure 5(b)], primarily due to the loss of chlorophyll but also due to changes in berry optical properties (becoming more translucent over time) [16]. Thus, ANTH_RG could also be a useful indicator for white grape cultivars. It is interesting to note that the fluorescence emission index SFR, thanks to its independence of excitation screening, was strikingly similar for both red and white grape cultivars. The behaviour of anthocyanin-related indices in Chardonnay [Figure 5(a) *vs*. Figure 5(b)], which is devoid of anthocyanins, showed that FERARI is noisier than ANTH_RG, displaying larger variations and larger confidence intervals, as was expected from the above-described tests on signal sensitivity.

### 3.3. Estimation of the Contribution of Red and Green Berries to Multiplex^®^ Indices (P-model)

**Figure 8.**
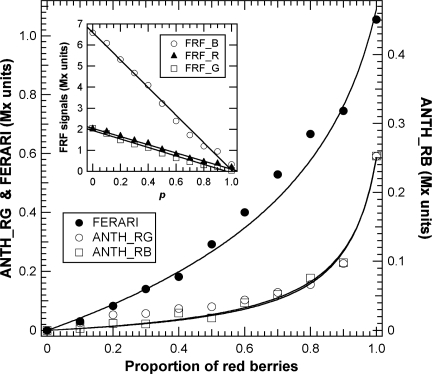
Multiplex^®^ signals and indices as a function of the proportion of red berries (*P*-model). Linear fit for the three FRF signals are shown, all r^2^ = 0.99. ANTH_RG and ANTH_RB indices where normalised by subtracting the constant log(*γ_RG_*) and log(*γ_RB_*), respectively. For the fits of ANTH indices, see text.

In previous publications on the application of the chlorophyll fluorescence screening method for grapes [16,18,29], the simple influence of green berries on the overall optical signal was not analysed. This type of influence on the chlorophyll fluorescence screening method is illustrated in Figure 8. Two populations of berries were mixed, including both red berries chosen individually with the Multiplex^®^ to have the same anthocyanin content and green berries devoid of anthocyanins.

Thus, if we name *p* the proportion of red berries, FRF signals should be proportional to the sum of the fluorescence of green berries having “naked” chlorophyll, 1 − *p*, and red berries, *p*, in which chlorophyll is screened (Figure S2). For green excitation, this behaviour can be described by the equation:

(6) $$\text{FRF}\_G=\gamma_{G}\left[\left(1-p)+(p\;T_{G}\right)\right]$$

where *T*_G_ is the apparent transmittance of the skin for green light before reaching the chlorophyll of red berries and *γ_G_* is a constant. The transmittance for green berries (1 − *p*) is assumed to be 1. Indeed, all three signals (FRF_B, FRF_G and FRF_R) declined linearly with the increasing presence of red berries (Figure 8, insert). This trend occurred during véraison at the level of each cluster or for samples of 200 berries from the vineyard. For all three wavelengths, this simple model showed a very large coefficient of determination, r^2^ = 0.99, between the observed and predicted values, so *p* could be estimated with an error between 1 and 4%. The root mean square error (RMSE) for *p* estimation was 0.029, 0.037 and 0.028 for FRF_R, FRF_G and FRF_B, respectively.

ANTH indices were less affected than FERARI, as expected, but they changed significantly above *p* = 0.5 (equivalent to the half véraison stage) [5] (Figure 8). Combining Equations (1) and (6) and using the corresponding suffixes, R, G and B for red, green and blue excitation light, respectively, the equation for the effect of *p* on ANTH indices will be:

(7) $$\text{ANTH}\_\text{RG}=\text{log}(\gamma_{\mathrm{RG}}\left[\left(1-p\right)+p\;T_{R}\right]/\left[\left(1-p\right)+p\;T_{G}\right])$$

(8) $$\text{ANTH}\_\text{RB}=\text{log}(\gamma_{\mathrm{RB}}\left[\left(1-p\right)+p\;T_{R}\right]/\left[\left(1-p\right)+p\;T_{B}\right])$$

and for FERARI from Equations (4) and (6):

(9) $$\text{FERARI}=-\text{log}(\gamma_{R}\left[\left(1-p\right)+\left(p\;T_{R}\right)\right])$$

The *γ_RG_*, *γ_RB_* and *γ_R_* are constants, and *T*_R_ and *T*_B_ are the apparent transmittances of berry skins before chlorophyll is reached. The fits of these equations to experimental data were very good, with a standard error smaller for ANTH_RG (0.0084) than for ANTH_RB (0.019) and FERARI (0.047) (Figure 8).

### 3.4. Calibration of Multiplex^®^ Indices for Skin Anthocyanins (A-model)

**Figure 9.**
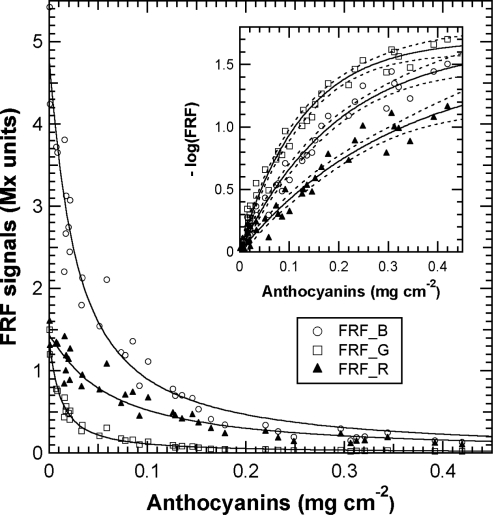
Multiplex^®^ signals used for the calibration of skin anthocyanins (*A*-model). Each point represents a Multiplex^®^ measurement (for signal nomenclature, see Table 1) performed on a 19-berry sample of Pinot Noir or Pinot Meunier (no distinction between cultivars). Anthocyanins were extracted from the corresponding berry skins (see Experimental Section). The transformation of the signal according to the Beer-Lambert law is presented in the insert.

The effect of anthocyanin screening in red berries now needed to be quantified and expressed in usual skin anthocyanins content units. Towards this goal, we analysed a series of berry samples chosen for their increasing redness with the Multiplex^®^, followed by the total extraction of the skins as done in [16–19]. The decrease in FRF signals that depends on the increase in anthocyanins in the skin, without the effect of the presence of green berries, is presented in Figure 9.

The chlorophyll fluorescence screening method postulates [27] that the FRF_G signal should be attenuated by the presence of anthocyanins in accordance with the Beer-Lambert law:

(10) $$\text{FRF}\_G=\gamma_{G}10^{-{ɛ}_{G}\;\mathrm{anthocyanins}}$$

where ɛ_G_ is the apparent absorptivity of anthocyanins in the green, and *γ_G_* is a constant. Therefore, the inversed and log-transformed FRF signal should be linearly related to anthocyanins content. From the insert in Figure 9, where normalised (division by the constant *γ_G_*), transformed signals are presented, it can be seen that this is not the case. Although the order of the absorptivity coefficients for anthocyanins was preserved (for malvidine-3-glucoside 24,590, 12,290 and 70 M^−1^ cm^−1^, for 516, 470 and 635 nm, respectively), the relationship was not linear and the absorptivity decreased with anthocyanin content. The transformed red-excited FRF signal, −log(FRF_R), which is the FERARI index, increased much more rapidly than expected from its *in vitro* absorptivity coefficient (anthocyanins do not absorb red light), but this signal was the closest to a linear dependence. In contrast, the low and constantly changing *in vivo* absorptivity of anthocyanins, obtained from transformed green and blue excited FRF (Figure 9, insert), can probably be explained by the overlap between anthocyanins and chlorophyll in the grape skin [16]. Indeed, unlike the situation in leaves, where a good separation exists between the proximal layer of epidermal cells and a distal layer of chloroplasts along the pericline cell wall of the first layer of mesophyll cells [36], in grape berry skins the first hypodermal cell layer containing anthocyanins already contains some chlorophyll. Therefore, the chlorophyll fluorescence screening method, which works very well for flavonols in leaf epidermises and probably also for flavonols in the berry epidermis [37], must depend on the small difference of 10 to 20 μm between the maximum in anthocyanins and chlorophyll distribution, the former preceding the latter in the berry hypodermis [16]. As discussed in [16], the decrease in the absorptivity of anthocyanins *in vivo* can also be due to the increase in skin pH during maturation. All these observations explain why the best fit of the data was obtained with a negative exponential function

(11) $$-\text{log}\left(\text{FRF}\_G\right)=A[1-\text{exp}\left(-a_{G}\;\mathrm{anthocyanins}\right)]$$

where A and a_G_ are constants of the fit. The constant A is the maximal value at infinite anthocyanin content, so we kept it the same for all three signals. This function can also be used to define the FRF’s decay with increasing anthocyanins. From Equations (10) and (11), we obtain the following power and exponential decay function for FRF_G:

(12) $$\text{FRF}\_G=\gamma_{G}10^{A[\text{exp}(-a_{G}\;\mathrm{anthocyanins})-1]}$$

which not only fits the signal best (Figure 9) but can now be used to derive the response function for ANTH_RG from the two signals decay functions and Equation (1):

(13) $$\text{ANTH}\_\text{RG}=A\left[\text{exp}\left(-a_{R}\;\mathrm{anthocyanins}\right)-\text{exp}\left(-a_{G}\;\mathrm{anthocyanins}\right)\right]+\text{log}(\gamma_{\mathrm{RG}})$$

**Figure 10.**
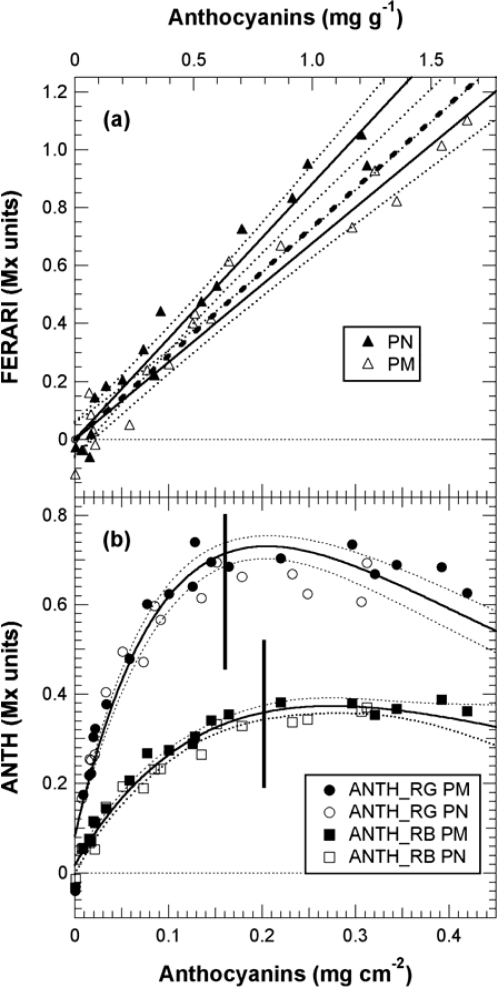
Calibration of the three Multiplex^®^ indices for the estimation of berry-skin anthocyanins (*A*-model). Indices were calculated from the signals shown in Figure 9. **(a)** Linear fits for FERARI are shown for Pinot Noir (PN) and Pinot Meunier (PM) individually or together (dashed line). **(b)** Fitted Equation (13) (response curve) for ANTH_RB (squares) and ANTH_RG (circles) for combined PM and PN data (full lines). Vertical lines indicate the limit used for numerical inversion and RMSE calculations for ANTH_RG (0.16 mg cm^−2^) and ANTH_RB (0.20 mg cm^−2^), respectively. Dotted lines are 95% confidence intervals. For presentation ANTH_RG and ANTH_RB indices where normalised by subtracting the constant log(*γ_RG_*) and log(*γ_RB_*), respectively.

**Figure 11.**
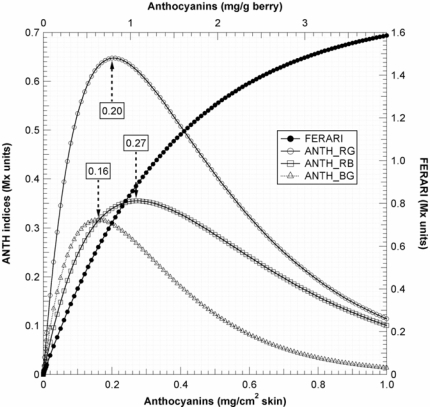
Simulated response curve for Multiplex^®^ indices covering the whole range of possible grape anthocyanin content. They were obtained using Equation (11) with red excitation for FERARI, and using Equation (13) for ANTH_RG, ANTH_RB and ANTH_BG with their respective excitations. The parameters of the equations were derived from the present calibration (data Figure 10). Maxima that can be attained by the three indices are indicated on the graph in mg anthocyanins per cm^2^ of skin.

Thus, in Figure 10(b), the ANTH indices measured during calibration were fitted with the respective forms of Equation (13). Using Equation (13) and the fit parameters of the calibration, A, a_R_, a_B_ and a_G_ (Figure 9, insert and Figure 10(b)), we could simulate the response curves for ANTH_RG, ANTH_RB and ANTH_BG for the full range of anthocyanin content expected for any grape cultivar (0 to 1 mg cm^−2^, equivalent to 0 to 4 mg g^−1^, Figure 11). One can see that the ANTH indices have two ranges of response to anthocyanin content, which increase in one range and decrease in the other, separated by a maximum (Figure 11). Response curves in Figure 11 can be used to derive graphically (numerically) anthocyanin content from any index value. The first range is delimited by the maxima at 0.2, 0.27 and 0.16 mg cm^−2^ of skin anthocyanin content for ANTH_RG, ANTH_RB and ANTH_BG, respectively. In addition, for the first range of the response curve, we derived polynomial functions by fitting the inversed response curves anthocyanins *vs*. Multiplex^®^ indices. Therefore, the following fourth-order polynomials can be proposed for ANTH_RG until a first limit of 0.16 mg cm^−2^ (equivalent to 0.6 mg g^−1^) (to avoid the flat range around the maximum)

(14) $$\mathrm{anthocyanins}=0.0567\;\text{ANTH}\_\text{RG}+0.688\;{\text{ANTH}\_\text{RG}}^{2}-2.05\;{\text{ANTH}\_\text{RG}}^{3}+2.28\;{\text{ANTH}\_\text{RG}}^{4}$$

and for ANTH_RB until a first limit of 0.2 mg cm^−2^ (equivalent to 0.8 mg g^−1^)

(15) $$\mathrm{anthocyanins}=0.181\;\text{ANTH}\_\text{RB}+2.33\;{\text{ANTH}\_\text{RB}}^{2}-12.3\;{\text{ANTH}\_\text{RB}}^{3}+26.1\;{\text{ANTH}\_\text{RB}}^{4}$$

to estimate skin anthocyanin content from Multiplex^®^ indices. The RMSE for anthocyanin estimation was very similar, 16 μg cm^−2^ (20%) and 16 μg cm^−2^ (16%), with an r^2^ of 0.90 and 0.94 for ANTH_RG and ANTH_RB, respectively. For Pinot Noir and Pinot Meunier, which have quite different skin characteristics [38], individual fitting of the response curves [Equation (13)] was not significantly different for ANTH_RG and ANTH_RB (data not shown) [cf. Figure 10(b)].

In contrast, the FERARI index could be used in the entire range up to 0.45 mg cm^−2^ (1.8 mg g^−1^) [cf. Figures 10(a) and 11] but with a maximum error up to 13% and an r^2^ of 0.96 for both PM and PN (RMSE = 29 and 21 μg cm^−2^, respectively). The differences in the slope of the FERARI calibration curves for the two varieties are trivial and can be explained by the larger average berry size for Pinot Meunier (2.25 g) than for Pinot Noir (2.05 g) later in the season. Pinot Meunier berries will give a larger fluorescence signal because they will be closer to the Multiplex^®^’s detectors. In the future, we recommend the use of a measurement geometry that will keep the proximal side of the berry (and of the cluster) at a constant distance from the detector. FERARI data can then be transformed into skin anthocyanin content based on the surface (mg cm^−2^) by using the inverse of the linear fit of Figure 10

(16) anthocyanins=FERARI/2.9

when considering both cultivars together with an r^2^ of 0.93 and an RMSE of 42 μg cm^−2^ (17%). The latter figure was much larger than for individual cultivars or for ANTH indices, but the index was validated for a much wider range (0 to 0.45 mg cm^−2^–1.8 mg g^−1^) (Table 3).

For ANTH_RG, different limits have been described previously using three different fluorometric devices: (1) using a spectrofluorometer with a limit at 300 nmol cm^−2^ skin equivalent to 0.15 mg cm^−2^ skin and 0.55 mg g^−1^ berry [16], (2) using a fluorescence imager (0.25 mg cm^−2^–1 mg g^−1^) [17], or (3) using the “leaf clip” Dualex-ANTH on peeled skins (0.3 mg cm^−2^–1.2 mg g^−1^) [18]. The limit attained here of 0.45 mg cm^−2^ (1.8 mg g^−1^) with the FERARI index based on a weakly absorbed wavelength is sufficiently high for the viticultural practice in Champagne (cf. below) and probably other regions. The limit of 0.2 mg cm^−2^ (0.8 mg g^−1^) attained using the first range of ANTH_RB is well adapted for a large number of table grapes cultivars but not for all winegrapes. Several red-winegrape cultivars, such as Nebbiolo (<0.8 mg g^−1^), have lower anthocyanin contents than Pinot Noir, even at full maturity [4]. For these cultivars, both the ANTH and FERARI indices are fully applicable using the first range of the response curve (the rising part in Figure 10). Many other cultivars (Cabernet Sauvignon, Merlot, Syrah) have even higher anthocyanin content values than Pinot Noir, so they will be in the second range of the response curve where ANTH decreases with increasing anthocyanins (Figure 10). However, ANTH indices could also be used in this second range to quantify anthocyanins using a polynomial function, but they will have to be calibrated with the larger skin anthocyanin content of appropriate cultivars in the future. It should be mentioned here that many other red fruits (apples, pears) have one order or magnitude less anthocyanins in their skins [20,39], so the first range of the ANTH response curve is well adapted to them.

### 3.5. Combination of the P-Model and the A-Model for the Estimation of Half-Véraison Date

**Figure 12.**
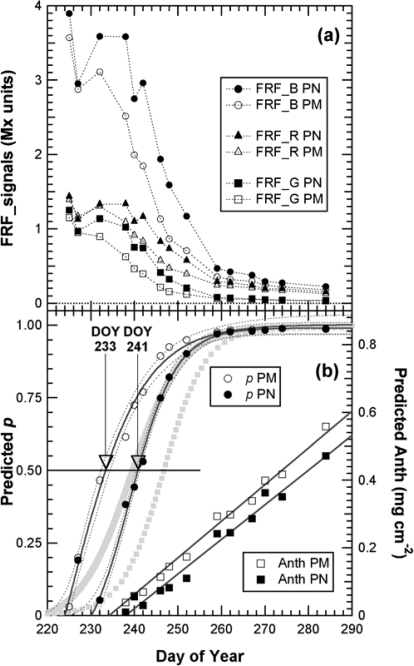
**(a)** Changes of Multiplex^®^ signals during the maturation of grapes at Fort Chabrol. For signal nomenclature, see Table 1. Mean Multiplex^®^ signals for 40 marked clusters per cultivar, Pinot Meunier (PN) (plain markers) and Pinot Noir (PM) (empty markers). **(b)** Kinetics of the proportion of red berries (*p*) and skin anthocyanin content (Anth) during grape maturation obtained by deconvolution of Multiplex^®^ signals (see text for details). Sigmoid fits for *p* and linear fits for anthocyanins are presented for each cultivar. The sigmoid fits to ANTH_RG data (from Figure 5) are presented in grey heavy lines for comparison.

We can now address the question of a combined *P* and *A* model to deconvolute both the proportion of red berries and anthocyanin content from the kinetics of Multiplex^®^ indices recoded in Figure 5. A combination of the basic equations of the two models, Equations (6) and (12):

(17) $$\text{FRF}\_G=\gamma_{G}\;\{(1-p)+p\;10^{A\left[\text{exp}\left(-a_{G}\;\mathrm{anthocyanins}\right)-1\right]}\}$$

could not produce a viable solution. Therefore, we decided to fit the FRF decays (Figure 9) by a function that can be inverted and can still satisfy the quality of fits obtained by Equation 12 (tested by comparing the Chi^2^, not shown). That new function was a hyperbolic function of the type f(x) = a/(b + x^1.1^). If we include this empirical hyperbolic function for each FRF signal, using the usual suffixes R, G and B to corresponding to Equation 6 of the *P*-model, we obtain the following system of Equations:

(18) $$\text{FRF}\_R=\gamma_{R}[\left(1-p\right)+p\;\alpha_{R}/(\beta_{R}+{\mathrm{anthocyanin}}^{1.1})]$$

(19) $$\text{FRF}\_G=\gamma_{G}[\left(1-p\right)+p\;\alpha_{G}/(\beta_{G}+{\mathrm{anthocyanin}}^{1.1})]$$

(20) $$\text{FRF}\_B=\gamma_{B}[\left(1-p\right)+p\;\alpha_{B}/(\beta_{B}+{\mathrm{anthocyanin}}^{1.1})]$$

with the six parameters, α and β coming from the calibration of the *A*-model (Figure 9). The constants γ were the values of the signals of green berries. For each date and cultivar (40 PM and 40 PN clusters), pairs of *p* and anthocyanins were calculated using any of the possible three pairs of excitation wavelengths [Equations (18) to (20)]. The solution for the red and green pair is shown in Figure 12(b). The solutions for the other two pairs were similar (cf. supplementary data, Figure S3). The proportion of red berries was fitted by a sigmoid function [Figure 12(b)]. The half-véraison, characterised by *p =* 0.5 (50% red berries), occurred at DOY 233 for Pinot Meunier and at DOY 241 for Pinot Noir. The confidence interval for this estimation was ±1 day. We would have made an error of seven days (a whole week) if we had based the estimation only on the direct ANTH_RG index. The model estimates the half-véraison date more accurately; therefore, the harvest date can be forecast more reliably [5]. The period from the first red berry in a cluster (véraison start; stage BBCH 81) to fully red clusters (hereafter DOY 260; véraison end; stage BBCH 85) can last more than 20 days (Figure 5). Therefore, it is very difficult to define half-véraison with precision by simple visual observation. This growth stage is important and often used to adjust viticultural practice (pest treatments, cluster thinning, addition of growth regulators). In addition, the deconvolution of *p* and anthocyanins (Figure 12) shows that the anthocyanin increase was rather linear during the season (r^2^ = 0.99 and r^2^ = 0.98 from DOY 232 and DOY 238 on for PM and PN, respectively) (Figure 12), unlike the increase indicated by the ANTH and even the FERARI index (Figure 5).

The model was validated on 200-berry samples from commercial blocks for which photographs and visual estimation of *p* were available (Figure S2). The combined *P* and *A* model was applied to twenty independent samples. A RMSE of 0.036 (7%) was obtained for the *p* estimation. The following characteristics of the linear regression (not shown) between the observed and estimated *p* were found: slope = 0.947 ± 0.0378, intercept = 0.049 ± 0.018, r^2^ = 0.988 and p < 0.0001 (extremely significant). Of the six parameters of the model (for two wavelengths), the two γ constants seem responsible for most of the uncertainty (data not shown). These constants depend on the instrument function (intensity of excitation light, sensitivity of detectors) that is almost eliminated by the use of the blue standard but also on chlorophyll content [16], on chlorophyll fluorescence yield (the sample should be under similar light and temperature conditions) and on the distance of measurement (Table 2), which should change as little as possible between the calibration and sample measurements. To that aim, we propose that grids or windows be used in the future to fix the measuring distance.

The decrease in skin chlorophyll content during maturation, attested by the decrease of SFR (Figure 5), was not taken into account. Our attempt to include this component in the model failed (not shown), possibly because it might be smaller than, or compensated by, the chlorophyll-overlap effect. Indeed, as explained above, the accumulation of anthocyanins produces a double exponential attenuation: the first from anthocyanins absorption and the second from the decreased chlorophyll excitation due to an anthocyanins-chlorophyll overlap [16]. Our goal was to produce a practical model to obtain *p* by an analytical, or at least a numerical, inversion. It was successfully tested for *p* and so it is useable in this respect. In addition, the model calculates anthocyanin content, but it was not tested for anthocyanin content explicitly because Fort Chabrol data were followed non-destructively and the 200-berry samples extracted by the standard wet chemistry method yielded incomplete extraction (cf. below).

### 3.6. Application to Viticultural Practice

Armed with calibrated indices and a model for the deconvolution of the contribution of green berries, we could address an example of practical large-scale application of the Multiplex^®^.

#### 3.6.1. Multiplex Method Compared to Standard Wet Chemistry

A very large variability exists at the cluster level [40] (Figure 5, confidence interval) or block level [19]. Thus, the usual practice in viticulture is sampling 200 berries to assess grape maturity [34]. We wanted to test whether Multiplex^®^ measurements on clusters *in situ* (in vineyard blocks) can be used, as shown in Figure 5 for the Fort Chabrol experiment, to replace berry sampling for block characterisation. We thus compared cluster measurements in the field to the 200-berry sample measurements in the laboratory and the wet chemistry extraction of these samples.

**Figure 13.**
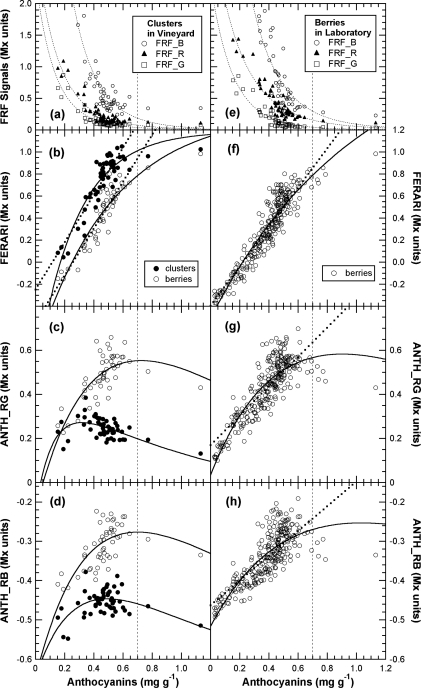
Comparison of Multiplex^®^ indices and wet chemistry estimation of anthocyanin content in Champagne region vineyards. **(a,b,c,d)** Blocks for which both 200-berry samples (laboratory) and 100-cluster samples (vineyard) were available. **(e,f,g,h)** 200-berry samples from 40 blocks sampled 6 to 8 times during the season (n = 273). The vertical dotted line indicates the limit of anthocyanin content attained in 2007 [19] and the maximum anthocyanins at the official end of harvest in Champagne in 2008. Means of Multiplex^®^ FRF signals recorded on 100 clusters in vineyard blocks (a) and on 200-berry samples in the laboratory (e) from the same blocks (n = 53). Signals (a,e) were fitted with Equation (12). Indices were fitted with Equation (13) [full lines in (b–d) and (g–h)] or with linear equations [broken lines in (b,f,g,h)].

FERARI values obtained on clusters were systematically larger than that obtained on 200-berry samples but with the same slope of the linear fit [Figure 13(b)]. It was the opposite for the ANTH indices, in which the indices for the clusters were smaller [Figures 13(c,d)]. The common origin resides in the systematically smaller FRF_R signal recorded on clusters compared to berries (Figure 13). In this part of the study, berries were not oriented on trays, so the side of the berry less exposed to sunshine and having less anthocyanins [41] will also be sensed by the Multiplex^®^. This orientation will increase the FRF signal, as will the unscreened chlorophyll present on the scar left by the pedicel removal. Finally, berries are usually sampled from all parts of the clusters, even from smaller, unripe, secondary clusters of the vine.

The coefficient of determination for FERARI obtained in the field on clusters against wet chemistry estimation of block anthocyanins [Figure 13(b)] was reasonably good (r^2^ = 0.81) considering the difference in sampling protocols (100 clusters *vs*. 200 berries). This result is the consequence of a good correlation between the 100-cluster and the 200-berry samples measured by the Multiplex^®^, although the relationship might not be linear (second order polynomial fit, r^2^ = 0.91) (not shown). Thus, the 200-berry sample extracts [Figure 13(b)], after division by the surface-to-mass ratio (SMR) (Figure 4), can be used to derive the formula for skin anthocyanin content based on the surface (mg cm^−2^) from clusters FERARI by the inversion of the linear fit of Figure 13(b):

(21) anthocyanins=(FERARI−0.2)/6.7

By comparing Equation (16) to Equation (21), it is obvious that the standard wet chemistry used by the winery is 2.3 times less efficient in extracting anthocyanins (proportionality factor 2.9 *vs.* 6.7). This result explains why the maximum of the ANTH_RG function (Equation (13)) for clusters is 0.32 mg g^−1^, corresponding to 0.08 mg cm^−2^, (Figure 13) a 2.3 smaller value than for the calibration in Figure 10 and Figure 11. This finding illustrates the complexity of using a reference method to calibrate optical signals.

All three Multiplex^®^ indices obtained in the laboratory on the 200-berry samples (containing mixtures of red and green berries, Figure S2) could compete with wet chemistry for anthocyanin estimation [Figure 13 (f–h)]. Taking into account the uncertainty of the precision of the (reference) extraction method [5], it is difficult to decide which method is responsible for the dispersion of data points. Before the 0.7 mg g^−1^-limit, r^2^ was 0.66, 0.74 and 0.83 for ANTH_RB, ANTH_RG and FERARI, respectively [Figure 13(f–h)]. However, on whole clusters [Figure 13(b–d)], FERARI is more advantageous than ANTH_RB and ANTH_RG, which decline after full véraison has been attained (above 0.3 mg g^−1^ anthocyanins) because they are in the second range of the response curve (cf. Figure 10). For the latter, the dispersion is very large, allowing only an r^2^ of 0.44 (p = 0.002) for ANTH_RG to be attained. This negative relationship has been observed previously and encouraged Cerovic *et al.* [18] to propose an inverted ANTH_RG index. It is now clear that this behaviour is due to the difference in FRF-signal decays (Figure 9) and the shape of the response curve (Figure 10). Therefore, because a large number of clusters or berries must always be used to overcome the large heterogeneity (see above), the use of the FERARI index to assess the maturity of a block is the best alternative, as its sensitivity to measuring distance might be mitigated by the large number of clusters.

#### 3.6.2. Whole-block Maturation Kinetics and Vineyard Block Characterisation

Multiplex^®^ measurements on clusters in the vineyard block can thus advantageously replace berry sampling and laboratory work. Cluster samples typically produce compositional data that are closer than berry samples to that of the fruit at harvest [40]. Collecting whole clusters has the obvious advantage of representing all berry positions within a cluster, thereby accounting for the within-cluster variation in berry ripeness. For each date, we estimate the required time per block to be 15 min for 60 clusters. The latter figure was estimated from the maximal standard deviation of 100 clusters sampled in this study, which varied during the season (data not shown) and in the Fort Chabrol calibration (cf. error bars Figure 5).

**Figure 14.**
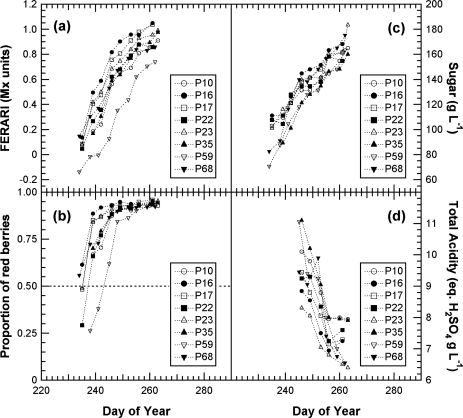
Complete vineyard block characterisation. Four blocks of Pinot Meunier (P10, P16, P17 and P35) and four blocks of Pinot Noir (P22, P23, P59 and P68) were chosen at random for this survey. Blocks were characterised by their accumulation of anthocyanins (phenolic maturation) using FERARI in the field **(a)**, the half-véraison date (from FRF_R & FRF_G in the field) **(b)**, sugar accumulation (by refractometry) **(c)** and decrease in total acidity **(d).** (c,d) Measured on the slurry from 200 berries.

The important half-véraison stage used to predict the harvest date could be estimated from FRF_R & FRF_G signals by applying the combined *P* and *A* model, as shown for Fort Chabrol data. Anthocyanin accumulation would be followed using the outputs of the *P*-model or, directly, using FERARI (Figure 14). This information might be sufficient for the qualitative selection of blocks and the forecast of logistic constraints regarding sizes of fermentation vats. Most advanced wineries would probably continue to use 200-berry sampling for sugar and total acidity estimation by classical methods (Figure 14). Figure 14 can thus be an example of a typical and complete report for maturity survey of a winery that can also be used for multi-annual surveys of the vineyard and adaptation of viticultural practices (pruning, thinning, fertilisation, among others).

A final issue should also be mentioned. For standard wet chemistry methods, the uncertainty of estimation is of the same order as the variation in anthocyanins content (0.05 mg g^−1^) during the last 20 days, and usually only one third of analysed blocks show a maximum in the maturation curve [5]. One of the reasons for this occurrence is that in later ripening stages, all the red colour of the berries could not be extracted by acidified solvents under standard procedures. The presence of coloured polymers (coloured tannin) has been detected previously in Cabernet Sauvignon [11] and Syrah [42] ripe berry skins. Knowing the difficulty of extracting and assessing these polymerised, often coloured, tannins [11], even an imperfect optical method that preferentially senses berry skin has practical potential. In addition to the method described here, other optical methods like the CIRG, based on L*a*b* colour parameters [43] or spectroscopy of reflectance in the UV, visible and NIR [14,21,39] have the same advantage. However, it seems that reflectance indices in the visible range saturate very early (above 0.05 mg cm^−2^, 0.18 mg g^−1^) due to a small reflectance [16]. Merzlyak *et al.* [39] proposed their method for apples in the range of 2.5 to 50 nmol cm^−2^ (0.00125 to 0.025 mg cm^−2^) well below that saturation limit. However, CIRG and NIRS warrant further comparison with the chlorophyll fluorescence screening method in the future.

## 4. Conclusions

The Multiplex^®^ sensor is very sensitive and sufficiently precise when accounting for temperature variations. Excitation ratios are robust at moderate distance variation, but individual signals and FERARI are affected by measuring distance. A new anthocyanins index based on red and blue fluorescence excitation (ANTH_RB) was tested in order to avoid early saturation of the FRF_G signal. Although it saturated later than ANTH_RG (Table 3), for low anthocyanin contents this new index was less useful in the field on oriented clusters, but could be beneficial on berry samples. The FERARI index was the least precise, but it had the largest range of application [up to 0.45 mg cm^−2^ (1.8 mg g^−1^) and probably even higher]. It can be used both on berries in the laboratory and on clusters in the vineyard.

The contribution of green and red berries (*P*-model) to the Multiplex^®^ signal was separated from the skin anthocyanin contribution (*A*-model). This method allowed us to calculate the half-véraison date and the kinetics of anthocyanin accumulation. The precocity of Pinot Meunier compared to Pinot Noir was confirmed and was determined to be 7 days (±1 day).

The Multiplex^®^ SFR index linked to the changes in skin chlorophyll content can be used to follow Chardonnay maturation in the absence of anthocyanins. There is as strong correlation between sugar accumulation and chlorophyll decrease. However, the robustness of this relationship and an absolute calibration for non-destructive prediction remains to be done.

With the introduction of the Multiplex^®^, the goal of implementing non-destructive, analytical methods directly applicable to grapes *in situ*, with the additional prospect of extending it later to overall vineyard mapping, is now within reach. Indeed, optical methods seem to be the only truly non-destructive way of measuring fruit constituents. In addition, due to their fast non-contact nature, these methods allow the analysis of a very large sampling population. Grape maturation, although very important, is not the only domain in which Multiplex^®^ indices can be applied. Other fruits (apples, pears, strawberries, *etc*.) and vegetables (tomato) have been considered and are currently being tested (FORCE-A, personal communication).

## Supplemental Information



## Figures and Tables

**Table 1.** Nomenclature of Multiplex^®^ signals. The name of each signal in the fluorescence excitation-emission matrix is defined by the abbreviation for its emission-light colour separated by the underscore sign from the abbreviation for its excitation-light colour: yellow (YF), red (RF) and far-red (FRF) fluorescence, excited by ultraviolet (_UV), blue (_B), green (_G) or red (_R) light. The central wavelength of each colour is indicated in brackets.

**Emission (nm)**	**Excitation (nm)**			
	**UV (373)**	**Blue (B) (470)**	**Green (G) (516)**	**Red-orange (R) (635)**
YF (590)	YF_UV	YF_B^a^	YF_G^a^	YF_R^a^
RF (685)	RF_UV	RF_B	RF_G	RF_R
FRF (735)	FRF_UV	FRF_B	FRF_G	FRF_R

a In the present configuration of Multiplex^®^, these signals are reflected light rather than fluorescence.

**Table 2.** Sources of measurement variability. Mean, standard deviation (SD) and percent standard deviation (%SD) are given for the major signal used (FRF_R, cf. Table 1) and for the Multiplex^®^ indices for anthocyanins (ANTH_RG) and chlorophyll (SFR_R).

**Source of variation**	**FRF_R**			**SFR_R**			**ANTH_RG**		

	Mean	SD	%SD	Mean	SD	%SD	Mean	SD	%SD
Repeatability^a^									
*Signal in mV*	2,297	10.7	0.5	2.578	0.015	0.6	0.629	0.003	0.4
*Standardised signal*	1.000	0.0047	0.5	1.000	0.0059	0.6	0	0.0026	−
Reproducibility^b^	0.902	0.016	1.8	0.984	0.008	0.8	−0.013	0.006	−
Temperature^c^	0.932	0.067	7.2	0.985	0.018	1.8	−0.010	0.014	−
Distance^d^									
Green berries	1.373	0.408	29.7	1.089	0.024	2.2	0.072	0.018	−
Immature red berries	0.918	0.356	38.8	0.948	0.019	2.0	0.493	0.021	4.2
Mature purple berries	0.142	0.039	27.7	0.701	0.028	3.9	0.601	0.007	1.2

a Thirty consecutive measurements on blue standard at 25 °C.

b Measurements on blue standard during the season, n = 11, temperature from 27 to 28 °C.

c The temperature range was 8 °C, obtained at different dates and different periods of the day (n = 29).

d Four measurements, ranging from 1 to 4 cm, using the standard distance from the detectors (10 cm).

**Table 3.** Range of applicability and precision of Multiplex^®^ indices for the estimation of anthocyanins using only the rising part of the response curve for Multiplex^®^ indices.

**Multiplex^®^ index**	**RMSE**			**Range**	

	**(μg cm^−2^)**	**(mg g^−1^)^a^**	**(%)^b^**	**(mg cm^−2^)**	**(mg g^−1^)**
ANTH_RG	16	0.063	20	0–0.16	0–0.6
ANTH_RB	16	0.063	16	0–0.20	0–0.8
FERARI^c^					
*Both cultivars*	42	0.166	17	0–0.45	0–1.8
*Pinot Meunier*	29	0.114	13	0–0.45	0–1.8
*Pinot Noir*	21	0.082	9	0–0.45	0–1.8

a Approximative values based on conversion figures of Figure 4.

b Percentage root mean square error in the middle of the range.

c FERARI was limited to the range of experimental data of this study.

## Supplementary Information

Figure S1. The study site.

Figure S2. Berry samples.

Figure S3. Predicted proportion of red berries.
