# Supplementary material for: Non-Destructive Optical Monitoring of Grape Maturation by Proximal Sensing
Source: Sensors (Basel). 2010 Nov 9;10(11):10040–68. doi: 10.3390/s101110040 (PMC3231004; doi:10.3390/s101110040)

Supplemental Information

Figure S1. The study site, vineyard Fort Chabrol, in Epernay. Pinot Meunier (PM), Pinot Noir (PN) and Chardonnay (CH) sub-blocks are indicated.

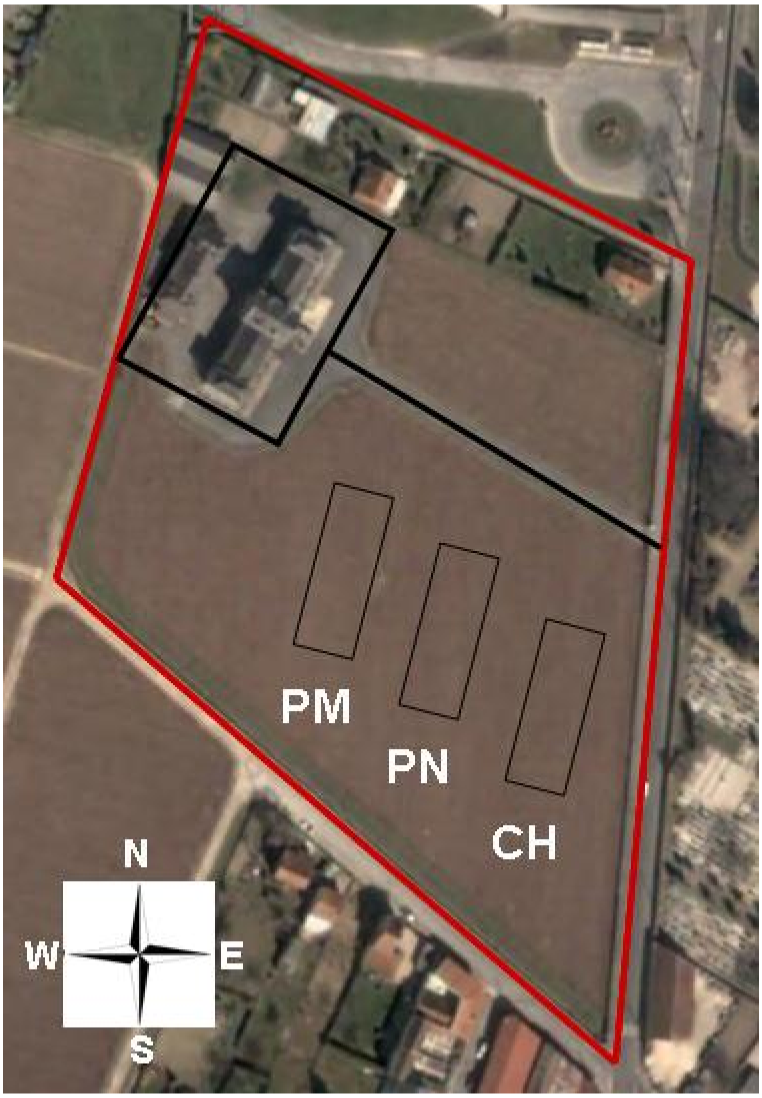

Figure S2. Berry samples: **(a)** for the effect of Multiplex-to-sample distance; **(b)** for *P*-model calibration; **(c)** for the validation of the combined *P*-model and *A*-model, and as an example of 200-berry samples; **(d)** 19-berry samples used for the calibration (*A*-model).

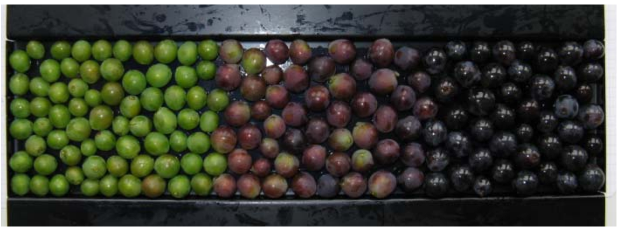

(a)

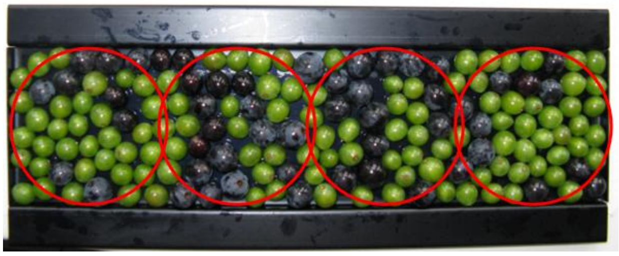

(b)

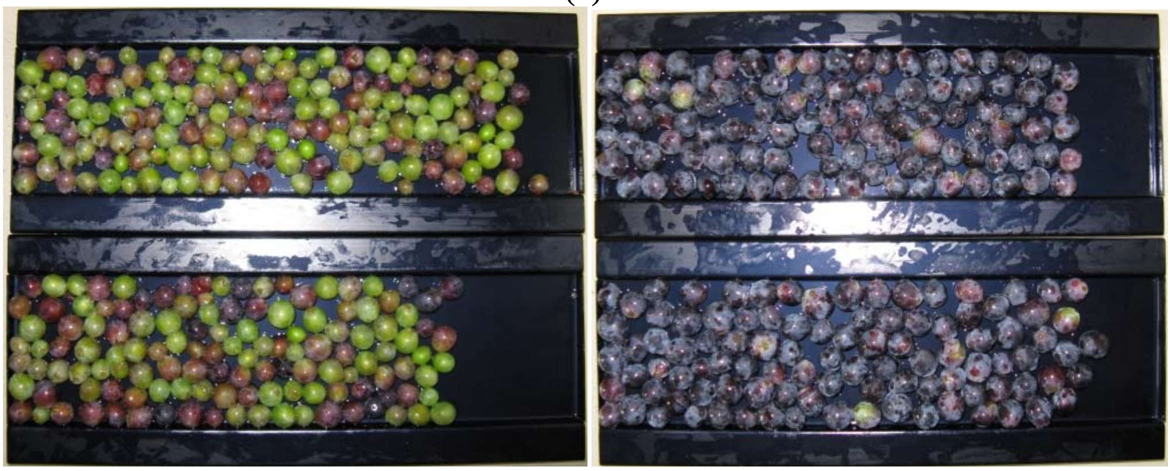

(c)

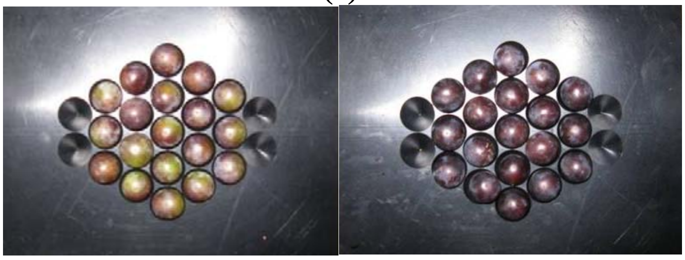

(d)

Figure S3. Predicted proportion of red berries and anthocyanin content using the combination of FRF_B and FRF_G (upper graph) or FRF_R and FRF_B (lower graph) signals in the combined *P* and *A* model. Sigmoid fit for *p* and linear fit for Anth.

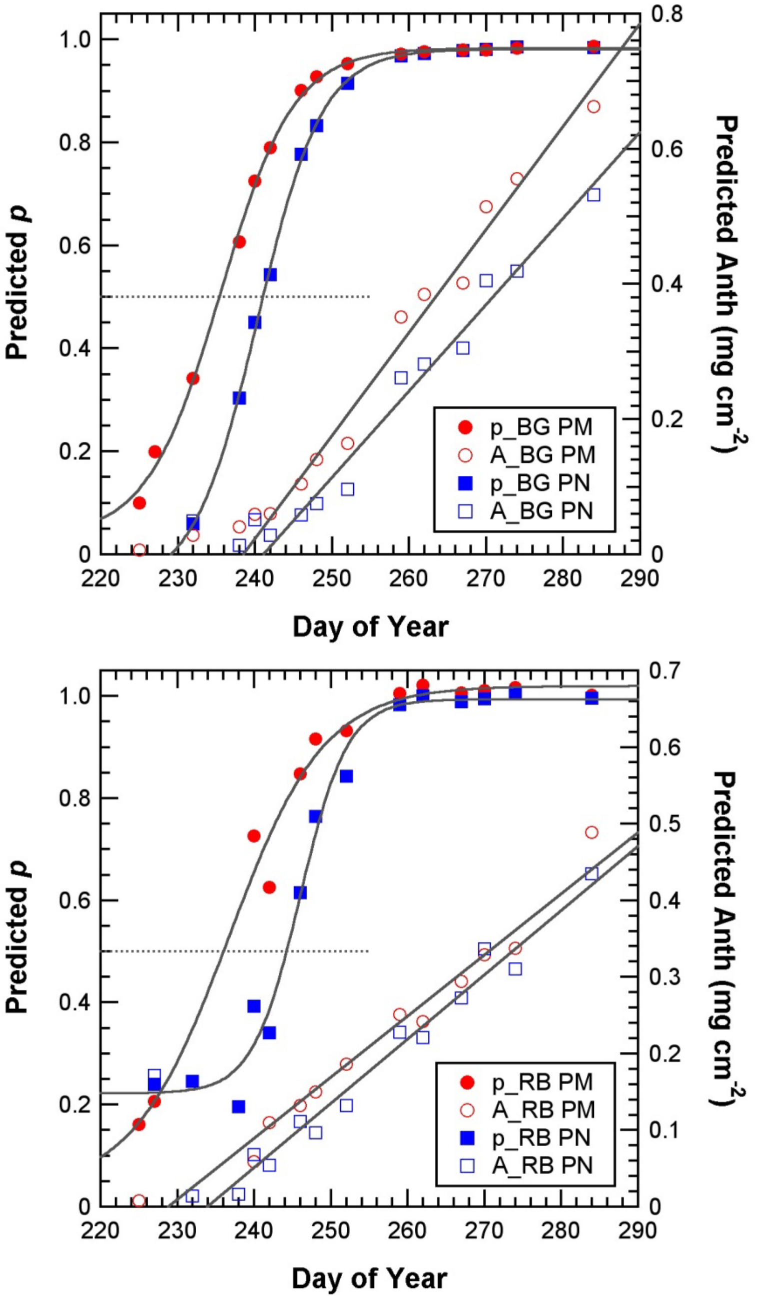

Supplement: Supplementary file 1 [file sensors-10-10040-s001.pdf]
